# The use of telehealth as a strategy to provide occupational therapy services in South Africa

**DOI:** 10.1177/10519815251374640

**Published:** 2025-09-12

**Authors:** Mogammad Shaheed Soeker, Aqeelah Amod, Erin Cuttings, Zahrah Fakier, Michelle Maas, Mika Stassen, Tatum Truter

**Affiliations:** 1 Occupational Therapy Department, University of the Western Cape, Cape Town, South Africa

**Keywords:** barriers, COVID-19, facilitator, healthcare, telemedicine, health professional

## Abstract

**Background:**

During COVID-19, healthcare professionals had to adapt their service delivery models to prevent virus transmissions using telehealth as a new service delivery model. Although many health professionals support telehealth, there is a void in the literature that focus on the use of telehealth in occupational therapy.

**Objective:**

This study aims to explore the experiences of occupational therapists in the public and private sectors regarding the use of telehealth in OT practice in South Africa.

**Method:**

Ten participants (occupational therapists who use telehealth as part of their interventions) participated in the study. The majority of the participants were 35 years of age, they were females and had 3.5 months of telehealth experience. The researchers made use of semi-structured interviews and used the process of thematic analysis to give rise to four themes.

**Results:**

Four themes surfaced which represent the experiences and perspectives of the occupational therapists utilising telehealth including the barriers and facilitators: The themes that became evident throughout the thematic analysis comprised (1) Enablers to the use of telehealth; (2) Barriers to telehealth in occupational therapy practice; (3) The use of education and research in strengthening telehealth as a strategy; (4) Strategies to enhance the use of telehealth in occupational therapy practice.

**Conclusion:**

The participants addressed the misconception that occupational therapists should not utilise telehealth due to the type of therapy provided, by elaborating on the influence of therapist adaptability and creativity on the integration of telehealth into their practice. Although telehealth minimises geographical barriers and costs associated with travelling, most participants had difficulty conducting assessments and navigating online platforms effectively from a therapist and client perspective.

## Introduction

A change in healthcare delivery known as telehealth uses electronic and communication technologies to deliver administrative, educational, and medical services remotely.^1^ As an aspect of Telehealth, telerehabilitation provides an integrated approach to patient treatment by allowing remote participation, evaluation, diagnosis and therapy. Nonetheless, issues with cost, effectiveness, practitioner skill, and legal frameworks continue to be raised. The task of recording and investigating the efficacy of telehealth services in clinical settings has been seen as challenging for occupational therapists (OTs). Some challenges include infrastructure restrictions, technological barriers, legal complexity, ethical issues, awareness gaps, financial limitations, and cultural rejection.^2^ Accepting and integrating Telehealth interventions—synchronous or asynchronous—into clinical practice is still a developing process.^3^

Occupational therapy (OT) is vital in helping individuals of all ages engage in meaningful occupations, contributing to their overall health and well-being.^4^ However, the emergence of the COVID-19 pandemic led to changes in healthcare service delivery to prevent virus transmissions, resulting in disruptions in traditional service delivery models.^5^ The latter resulted in restricting access to OT services, particularly for vulnerable populations who often live far from hospitals and rehabilitation centres. Healthcare providers were compelled to adopt Telehealth without adequate preparation or training.^6^ Telehealth in providing OT services represents a promising avenue for overcoming barriers to care and ensuring continuity of services in South Africa (SA). Telehealth offers numerous advantages, including time duration, enhanced accessibility, and increased patient satisfaction, yielding positive outcomes for patients and healthcare practitioners.^2^ In a study conducted by Breeden et al.,^7^ they indicate that occupational therapists had the view that telehealth programmes increased access to clients and facilitated the continuity of services. Furthermore, the results of a systematic review conducted by Feldhacker et al.^8^ reveal that there are strong to moderate evidence for the efficacy of telehealth services in occupational therapy, particularly for clients with neurological conditions.

The pandemic has underlined the importance of equipping individuals working from home with the necessary skills and resources to navigate the challenges of Telehealth.^5^ By embracing Telehealth into OT practice, high-quality, and accessible services to individuals across diverse settings may be ensured. Telehealth activities is an accepted form of intervention for all health professionals in South Africa and has been supported by the Health Professions Council of South Africa. Although many health professionals support Telehealth and its advantages, the lack of knowledge and understanding of the use of Telehealth is a concern among health professionals.^9^ Therefore, a need for research to be conducted on the usefulness of Telehealth strategies in OT within the South African context is needed. This will aid in informing the best practices, optimise service delivery, and improve outcomes for SA individuals and beyond.

## Literature review

### Implementation of telehealth

Certain first-world countries (e.g., the United States of America) have already utilised Telehealth in the more rural areas of the country before the COVID-19 pandemic, although this had not been widely implemented at the time.^10^ The United States of America saw an increase in the use of Telehealth from 0.1% pre-COVID-19 to 43.5% during COVID-19.^10^ Other countries, such as Australia, mainly utilised telehealth in rural areas before COVID-19 for specialist videoconferencing. Fisher and Magin^10^ further state that vulnerable individuals can access healthcare more easily through Telehealth to minimise the distance needed to travel to access healthcare providers, as well as individuals with low socioeconomic statuses. However, Gurupur and Miao^11^ state that implementation of Telehealth has been more challenging in rural areas, describing numerous challenges, including the complexity of using Telehealth with some users stating that Telehealth is too difficult to use. With large percentages of the population not having adequate access to technology and a lack of availability to reliable network connections, it can result in increased anxiety due to technological inexperience in rural communities.^11^ In Limpopo, South Africa, more than 80% of the population lives in a rural area.^12^ Therefore, due to the above-mentioned barriers, successful implementation of Telehealth in rural communities in a third-world country could be a demanding process.

### Ethics-related challenges in the use of telehealth

While all mediums of healthcare present their potential ethical challenges, Telehealth is often perceived to face more challenges due to its newer and less-studied implementation. Patients and healthcare professionals alike have raised concerns over the potentially reduced quality of care, the safe storage and record-keeping of data, and the potential for digital misuse including compromised confidentiality and improper consent practices. Mention has also been made about the relationship between the therapist and the client, as well as their overall level of satisfaction with the service delivered.^13,14^ Specifically, in the South African context, medical professionals have raised concerns over the risk of increased inaccuracy and lack of physical handling such a medium would require.^15^ These concerns increase individuals hesitating to make use of Telehealth-based services as a decrease in quality of care would be in direct violation of the ethical principle of “beneficence” and potentially decrease self-efficacy in practising professionals.^16^ Although the Health Professions Council of South Africa (HPCSA) has attempted to address these ethical challenges by compiling a Telehealth guideline, very few resources are available for professionals looking to explore this new medium of practice, and most ethical concerns remain persistent, unaddressed, and unexplored.

### Facilitators and challenges of telehealth in OT

Telehealth is described as an efficient and cost-effective tool to bridge the gap between served and underserved groups to access service delivery in the health sector.^17,18^ Little et al.^19^ further elaborated on the benefits of utilising Telehealth in intervention with autistic children including improved development of parental self-efficacy, increased collaborations; and convenience resulting from the use of Telehealth. Facilitators contributing to successful Telehealth implementation include administrative, interpersonal, family resources and clinic culture. Administratively emphasising the importance of providing practitioners with training to ensure successful implementation. Furthermore, Angell et.al.,^20^ correlate the barriers experienced to clinician's self-confidence towards challenges related to the successful conduction of OT in Telehealth.

### Occupational therapy experiences

Little et.al.^21^ confirmed that during the COVID-19 pandemic, OTs based in clinical paediatric settings were required to adapt their face-to-face manner of providing treatment and implement the use of Telehealth. Additional challenges include a decrease in the total hours charged for treatment and decreased referrals from doctors or paediatricians due to the decrease in access to clinics and hospitals during the pandemic. According to Sánchez-Guarnido,^22^ the COVID-19 pandemic has brought about great challenges within OT services, due to the lack of face-to-face intervention. This research found that the subsequent effects of the COVID-19 pandemic led to decreased in-person intervention, putting people at risk of relapse and declining mental health. Therefore, OTs have had to adapt to the use of online care service delivery, such as Telehealth services, allowing fair access and enriched opportunities to those who cannot access in-person consultations or intervention.^22^ According to Sánchez-Guarnido et al.,^22^ more research is needed regarding the effectiveness of OT Telehealth services and the impact of the COVID-19 pandemic on OT services within South African private and public practice. There is a gap in the literature regarding the experiences of the usefulness of Telehealth within OT practice, therefore highlighting the need to evaluate effectiveness, outcomes, service delivery, access and technological adaptations in Telehealth.^23^

## Aim

The study aimed to explore the experiences of occupational therapists in the public and private sectors regarding the use of Telehealth in OT practice in South Africa.

### Objectives

- To explore and describe the barriers to utilising Telehealth in OT practice.
- To explore and describe the facilitators aiding OTs to utilise Telehealth in OT practice.
- To explore the views of OTs regarding the use of Telehealth, as a form of evidence-based practice within the South African context.
- To explore possible suggestions to enhance the use of Telehealth in OT practice/services in the public and private sectors within South Africa.

## Methods

The study utilised the social constructivism paradigm, to seek an understanding of the subjective view of Telehealth experiences of OTs in the public and private practices of South Africa. The study used an exploratory and descriptive research design while utilising a qualitative approach.^24^ The exploratory and descriptive design enabled the researcher(s) to explore OTs’ perspectives on using Telehealth in OT and rehabilitation.

### Research setting, sampling and participants

This study was conducted online using the Google Meet and Zoom platforms to interview 10 participants, located in regions of the Western Cape and KwaZulu-Natal. Participants of this study, although all in private practice, varied in age and specialities within OT. These include mental health, intellectual disability, physical conditions and vocational rehabilitation (Appendix 1, Table 1-Demographic Table).

The sampling strategy used in the study was snowball sampling. Snowball sampling is a technique that requires existing participants to identify others they may know of,^25^ to gather perspectives on Telehealth use by OTs in South Africa. The researchers recruited 10 participants, to explore the use of Telehealth strategies among OTs due to limited theoretical knowledge about the topic. A sample of ten participants are regarded as appropriate for qualitative studies, as they focus is on understanding the depth of the phenomenon being explored.^26^ Participants were contacted electronically via WhatsApp, email, or telephone, and recruited from online sources like social media and through Google search. Participants in the study were chosen from individuals who were knowledgeable about the research topic. Potential participants were provided with information about the study before they were asked to consent to participate in the study. The inclusion and exclusion criteria for this study are indicated below (Appendix 1, Table 2 – Inclusion and exclusion criteria).

### Procedure

The researchers gained access to the contact details of participants by doing a Google search of OTs who use Telehealth in South Africa. Contact details of participants were also obtained through snowball sampling whereby each participant was asked if they knew of any OT who uses Telehealth that the research team could contact. If it was found that participants did not meet the inclusion criteria, they were informed and removed from the list of participants. The student researchers got into contact with the participants through email as well as through telephonic discussions. The six student researchers were final year students, studying towards a Bachelor's degree in Occupational Therapy. These students have had prior experience of conducting qualitative research, in particular, semi structured interviews with research participants. They provided the OTs with an information sheet containing information about the study, consent forms as well as the ethics clearance letter. The researchers used online semi-structured interviews, ranging between 40 and 75 min each, to collect the data. The semi-structured interview consisted of two researchers meeting one participant at a time where the researchers interviewed by asking the participants questions using a pre-established question list (See Appendix 2). The semi structured interview guide was developed by focusing on the research question and objectives of the study. The questions aided in understanding the experiences of occupational therapists regarding the use of telehealth services. The semi structured interview guide was reviewed by the research team. Currently there is no formal telehealth course offered at universities in Cape Town. The participants could decide the date, time, location and online platform of their choice to participate in the interview so that it was most convenient for them. A total of 10 interviews were conducted until saturation occurred, with each participant being interviewed once. Individual interviews were recorded and transcribed by the researchers. The researchers ensured that they monitored the participants’ body language and facial expressions to determine whether any discomfort arose from the discussion. Before analysing the data, each audio recording of the interview was transcribed verbatim by the researchers present in the interview. The questions that were asked focused on the participants’ (OTs) experiences of using Telehealth within South Africa. In total, 4 questions were asked, each with three probes to provide further clarity if needed. The questions were open-ended so that the participants could express their opinions freely about the topic. The participants were able to answer the questions in detail and sufficiently, thus the predetermined question list proved to be sufficient. Member checking was done by sending the categories and themes to each participant via email where they had the opportunity to comment on these themes and categories.

### Analysis

The researchers selected the use of a thematic analysis to analyse their data. A thematic analysis was utilised in this study, to identify, analyse and depict certain themes and/or patterns within this qualitative research study.^27^ The data analysis strategies advocated by Braun and Clark,^27^ were used in the study. Once all researchers completed the transcript coding process, all relevant and applicable themes and categories were formulated based on similarities and trends within data analysis.

Trustworthiness in the current study was ensured through credibility, transferability, dependability and confirmability. Credibility was ensured through reflexivity and member checking, and reflexivity was ensured using a field journal to practise reflexivity and to note the important observations within the interviews.^28^ Reflexivity aided the researchers in understanding how their preconceived ideas and personal circumstances influenced the outcome of the study. Fieldwork notes were saved by the researcher(s) for each interview through voice notes and by typing them out in the form of messages. The researcher(s) utilised member checking to ensure that the findings related to the study were a true reflection of the information provided by the interviewees. Member checking has been conducted with a few of the participants to verify the findings of the study. Transferability was ensured through the detailed description of the research methods such as qualitative research used in the study and contexts of the research participants. Dependability was ensured through the dense description of the study to the participants as well as the use of peer examination, whereby research students presented the findings of the study to their research supervisor and peers. This process enabled the students to obtain feedback about the accuracy of the findings of the study. Confirmability was informed using a reflexive journal as described previously and the use of a confirmability audit trail.

## Ethics

The research study commenced after obtaining approval from the Biomedical Research Ethics Committee of a university in the Western Cape. Various therapists were contacted via email and/or telephone calls to enquire about their interest and availability regarding the study. Information was sent electronically to the participants to ensure informed decision-making. This included our study aim, objectives, inclusion criteria, and expectations regarding participation. Participants were ensured confidentiality, and no identifying information was shared outside of research purposes. Additionally, the researchers reinforced the concept of non-maleficence by giving truthful accounts of the participants’ experiences and providing them with counselling resources for any emotional challenges that may have arisen as a result of the study. Finally, the participants had been informed that the study was being conducted voluntarily, meaning that they were under no obligation to assist us and that they could withdraw from the study at any time without consequences.

## Results

### Theme one (1): enablers to the use of telehealth

This theme discovered the various resources, experiences and means that aided OTs in utilising Telehealth as a means of intervention across virtual OT practice. Theme one addresses the objective that explores and describes the facilitators aiding OTs in utilising the medium of Telehealth in OT practice within the South African context.

#### Category one (1): overcoming one's fear of using telehealth as a health service strategy

This category was formulated based on the number of participants who had expressed their fear regarding the use of Telehealth. They mentioned fear of the unknown, lack of experience, and fear of doing things incorrectly as reasons they had avoided the service medium in the past. One participant said:

“People want the change, but they fear to take that change.” (P1)

While few participants had offered Telehealth services before the COVID-19 pandemic and subsequent lockdown protocols, the majority had overcome their fears to commence with Telehealth services out of necessity. They were motivated by the financial support it would offer and the desire to address a perceived void in the market, further emboldened by seeing other medical professionals offer Telehealth services as well as the ease and low-cost commitment with which they could set up their online practices. The participants said:

“COVID was a huge factor in pushing us into telehealth.” (P7)

“And I'm telling you once people get used to this, they would just want to do this because it's so convenient.” (P1)

#### Category two (2): telehealth service aids in the provision of occupational therapy services

This category was formulated based on the number of participants expressing their optimistic views and experiences using Telehealth as a tool within their OT Practice. Participants expressed their positive experiences referring specifically to convenience, accessibility, and flexibility as common words used. The majority of participants highlighted Telehealth as a valuable means to connect therapists and clients, overcoming physical, logistical, emotional, and geographical barriers. The participants said:

“It's been a tool for those who are living far away to have access, those whose pathologies are limiting their physical or emotional mobility, and to facilitate the logistical stuff much quicker, much more effectively.” (P9).

Participants elaborated on the accessibility that Telehealth provides for healthcare professionals and clients, despite the geographical, physical, emotional, or logistical barriers that may inhibit a client or therapist from attending physical therapy sessions, highlighting the view on how Telehealth has allowed for the expansion and provision of OT services virtually.

### Theme two (2): barriers to telehealth in occupational therapy practice

This theme explores the various difficulties of effectively delivering OT through telehealth. It covers technological challenges; the difficulties therapists face in conducting accurate assessments and treatment without physical presence; and client-related challenges. The theme addresses the objective of recognizing and understanding these barriers as well as providing insights into the difficulties therapists face in implementing Telehealth in OT practice.

#### Category one (1): technological challenges faced by the therapists and clients upon utilising telehealth

Multiple participants highlighted the challenges that were faced when utilising telehealth, this includes technological difficulties. Participants mentioned that the therapist or clients struggled to navigate various platforms, some lacked the necessary knowledge and skills needed to participate in online sessions, and age was brought up regarding the lack of knowledge as therapists or clients who were younger had more experience concerning working with technology. Participants expressed that specific clients with cheaper or older devices experienced poor quality network signals which affected the quality of assessments and interventions during online sessions, another factor being that the device did not meet the suitability for the platforms needed. One participant said:

“I don't know, the barriers I would definitely say is understanding that the person understands that it is a therapy service, that they know how to work their devices to be able to do this kind of thing” (P6)

Then network or connectivity-related issues were expressed by the participants as a barrier. Factors such as load-shedding, Wi-Fi, internet or software issues. Common issues brought up by participants included network issues that interfere with online sessions from either the therapist's end or the client's end, which has caused interference with discussions and interventions. The participants said:

“And then there's all the things of connectivity, data costs, electricity, load shedding.” (P6)

“So, if we're using Google Meet, if their connection isn't great, it often would work for a WhatsApp video call. If that doesn't work, I give them the option that we can either do a WhatsApp voice call or we can cancel the session.” (P10)

#### Category two (2): how telehealth impacts therapist assessments

Multiple participants expressed the limitations of utilising Telehealth that implicates assessing clients. Physical assessments were deemed more complicated as a trust was embedded in the client or the client's affiliate who was required to assess while listening to the instructions given by the OT. One participant stated that personal connection and personal engagement remained a barrier with Telehealth and thus impacted the assessment process as trust needed to be built.

“In terms of your relationship that you build with the clients. What I mean by that is body language that you pick up, being able to see the patient sitting in front of you. There is a lot of observation that goes into it, and even just seeing them walking in self-confidence. You don't see it over telehealth.” (P8)

“I don't think there's anything that can make it possible for me to fully assess someone online or to fully get a sense of their being, their behaviours and their body language” (P5)

#### Category three (3): client-related challenges that negatively impact the use of telehealth

OT practitioners in South Africa encounter various challenges with Telehealth services. A significant client-related issue is cost; many clients cannot afford the necessary technology and internet access, and smaller practices often resort to less effective, budget-friendly alternatives. Establishing therapeutic relationships is challenging without physical presence. As one participant mentioned:

“So, I think that's one of the main things about Telehealth, how that's really difficult to navigate is that personal connection that you actually don't have with the person.” (P6)

The informal setting of Telehealth can blur professional boundaries, leading to disruptions during sessions and concerns about confidentiality, especially if clients lack private spaces. Participant Five (P5) stated:

“Definitely, you don’t know who else is in the room, you also don't know who else is in the environment.” (P5)

### Theme three (3): the use of education and research in strengthening telehealth as a strategy

This theme describes the diversified experiences and efforts that assisted OTs in utilising telehealth as a means of intervention, through the exploration of education and research. Theme three addresses the objective of evidence-based practice as a means to improve greater telehealth implementation.

#### Category one (1): how therapists used education and research as a means of exploring telehealth in practice

This category was formulated based on the manner therapists had prepared themselves for Telehealth use including training they underwent and ways they had upskilled themselves, which was compounded by the lack of telehealth resources available to them. While there was the occasional course or workshop, it was rarely targeted towards OTs. The majority of participants highlighted the importance of undergoing their own personal research and exploration, which in turn, has aided or hindered them in improving their virtual abilities in telehealth practice. The participants said:

“There wasn't really any training on it, there was really no support in how to … I haven't seen many (courses), but the ones I have seen, there hasn't been much training on how to use telehealth most effectively.” (P9)

Despite the self-researching and self-learning around telehealth practices and platforms, a handful of participants believe that there is a lack of research regarding the use of telehealth, urging for the expansion of telehealth research. The participants acknowledge the self-researching involved in telehealth practice but continue to emphasise the need for more comprehensive research in telehealth.

“I think that there needs to be an opportunity to talk about how we're all using this medium.” (P3).

#### Category two (2): positive client outcomes

This category arose from therapists who have used telehealth with clients whose diagnoses vary. Most of the therapists stated that initially, especially during COVID-19, they would see any client online. However, they have since found that certain clients benefit more from telehealth whilst other clients benefit more from in-person therapy. The therapists used these findings, together with supporting literature about various diagnoses, to inform their future practice as they would recommend that a client be seen in person or over telehealth. The participants said:

“I think for clients who are very anxious, or who are anxious about going out into the world, that at least means an access point. It means we can really start a good relationship rather than them having to come in and that is such a big barrier that they don't even come in for initial sessions.” (P10).

Furthermore, they also found that children who have Autism Spectrum Disorder (ASD) tend to respond very well to telehealth.

“It is fun, it is also entertaining and so we did get that engagement. Especially kids on the spectrum – kids with Autism did brilliantly with online therapy, so well.” (P4).

#### Category three (3): telehealth on improving observations and assessments

Multiple participants mentioned that telehealth provides an opportunity to see the client's environment around them, limiting the need for the therapist to see the client's environment in person, saving time and costs for both the client and therapist. Participants were able to make use of Telehealth to see how their home is set up, in order to make the necessary adaptations. The participants said:

“You get to see the dynamics of the families, you hear things in the background, you see parent-child engaging a lot more, and you learn from there.” (P4)

“If we’re really looking at how we equip a child to play, learn and thrive in their environment, you are actually in their environment and it's really impossible to drive to each home if you have a practice as well. So that's quite amazing.” (P4)

Telehealth may also benefit the therapist in terms of doing certain standardised assessments. It was mentioned that telehealth provides the therapist with the time slots in video form, making it easier for the therapist to write out the assessment findings.

“The very first day and we took a video of that, and we can use that as measurement at the end as well. Even if you MODAPTS it, you put how many seconds you know, with the video you have immediately the seconds, you have the quality, you can see everything” (P2)

### Theme four (4): strategies to enhance the use of telehealth in occupational therapy practice

This theme focuses on strategies used by the participants to improve the efficacy of telehealth in OT. Subsequently, the strategies can contribute to the increase in OTs utilising telehealth as part of their intervention. Strategies include enhancing the potential of telehealth as a strategy of health care and promoting telehealth as a health-related strategy.

#### Category one (1): enhancing the potential of telehealth as a strategy for healthcare

To enhance the potential of telehealth as a strategy of health care, OTs expressed a need for formalising and improving guidelines in the use of telehealth for health professionals. Participants shared that the lack of clear guidelines for telehealth directly influences their confidence in performing telehealth services. The participants said:

“If there were clear guidelines for Do's and Don’ts, which there were at the beginning of COVID. However, nobody bothered to update it. Maybe that would also give people confidence in actually using it as an option.” (P5)

Exploring the use of physical resources and infrastructure to enhance the use of Telehealth services, all participants agreed that the lack of physical resources for both the client and therapist influenced the success of using telehealth services.

“Obviously the right technology. Maybe I could gain funding, for example, if it's an employer, let's say, to give someone the right laptop or phone or whatever to be able to access the service.” (P5)

#### Category two (2): promoting telehealth as a health-related strategy

The desire to improve telehealth utilisation in OT within South Africa brought about this category. The findings show that telehealth's potential is being recognised more, but they also point to a significant knowledge and education gap that affects both the public and OTs. Participants noted that many OTs may be using online resources without fully realising their potential, and many voiced a wish for greater awareness of and integration of telehealth into OT practices. A participant highlighted:

“I felt it is good for people to know just the scope of what one can do online.” (P3)

There was also a need for universities to include telehealth in their curriculum so that OTs of the future would be knowledgeable about this kind of service delivery. One participant said:

“Well, I would start with you guys as students. I would go straight to the universities. That's been on our plan for so long. Just so that as a student, as you enter this space, you’re already aware that this is an option for me.” (P4)

## Discussion

### Facilitators

Theme 1 discussed the various facilitators of telehealth use in the South African context, better understood with the help of the Layered Telemedicine Implementation Framework which highlights pre-existing determinants for successful telehealth implementation. Despite this, many participants had shared that they originally avoided telehealth as they lacked knowledge and experience regarding the service medium. A study conducted in 2016 found that few countries have conducted research regarding telehealth-based education and training and that no African countries had been represented in the published materials.^29^ This is concerning as it increases the chances of therapists conducting ineffective or even harmful therapy and because the education and training that has been made available is unlikely to be applicable in the South African context. However, following the COVID-19 pandemic some therapists had decided that the benefits of telehealth use ultimately outweighed the potential risks associated with practising via a largely untested service medium.

Based on the findings of our study, many participants described telehealth as convenient, flexible and accessible. According to Proffitt et al.,^30^ OTs work virtually with clients across varied settings, speaking to the accessibility, convenience and flexibility of telehealth. Not only does it allow OTs to reach clients remotely, where geographical barriers are a major limitation, but it also allows for flexibility in terms of reduced travel costs and travel time.^30^ In a South African context where socioeconomic status and resource-constraint reality are present, OTs need to be cautious when increasing virtual services country-wide, where resources may be scarce, despite the potential benefit of virtual service provision.^31^ This is validated when the participants mentioned the country's lower socio-economic status and connectivity issues. According to Rabe,^2^ although challenges remain, recent findings have shown that remote sessions are effective, feasible, and safe in South Africa, and encourage all healthcare professionals to become competent in utilising telehealth.

### Barriers

Theme two explored the several challenges of effectively providing OT via telehealth. Despite the mere preparation on behalf of the OT to ensure that their technology is up to date, or that the necessary Wi-Fi, data or network setups are in place for a smooth online session to take place; the clients’ accessibility to the same resources, also determine the quality of the session. Participants mentioned that clients from lower socio-economic areas without access to Wi-Fi or data, lack knowledge regarding technology utilization, and possess old-dated devices, impact the quality or performance of the online session. Furthermore, another study has shown the significant digital urban divide among the more developed and less developed communities, thus demonstrating the differences in the digital connectivity growth rate between the urban and rural communities. This affects their ability to acquire the necessary IT skills, and they struggle to afford mobile devices and other technology devices due to their low income.^32^ Thus, substantiating the mere fact that individuals from lower socio-economic areas in South Africa may lack the necessities that contribute towards telehealth.

There is a lack of research regarding the impacts telehealth displays upon collecting assessment data on clients within the South African context, however, according to Jacobs et al.,^33^ the study underlined the complexities of evaluation and the limitations of telehealth technologies within rural areas, that training would be required to ensure that the therapist can utilise the technologies to receive exceptional results. Moreover, the same study mentions that the validity and reliability of utilising standard assessments remains a question since standard assessments originally were constructed for in-person use.

Cost and accessibility issues are significant as lack of digital device availability, restricted internet and technology access, and unsatisfactory audio or visual quality^34^ hinder effective delivery, particularly for smaller OT practices that cannot afford premium platforms. Some participants felt that engaging in a virtual environment can complicate the formation of therapeutic relationships. In a study by McCoyd,^35^ it was found that therapists often felt that many of the qualities such as reading a client's body language were lost during the first six months of the pandemic transition to videoconferencing for therapeutic work. Additionally, professional boundaries and confidentiality concerns arise, with the need for therapists to be aware of the client's location before starting the telehealth session to ensure privacy and maintain trust.^36^

### Evidence-based practice

Theme three discussed how telehealth use can and will contribute to greater evidence-based treatment strategies for OTs. Many therapists found that telehealth in OT currently lacked the vital research and supporting resources needed to inform their evidence-based practice. According to Wamala & Augustine,^37^ telehealth is still considered to be in its infancy stage, therefore requiring further supporting tools and resources. Some participants claimed that this void made the transition from traditional therapy to online therapy difficult and gave them a negative outlook on the service medium. Mahtta et al.^38^ believe that telehealth research and support sources should be improved and available for healthcare professionals to ensure the effective use of this medium. Edirippulige^29^ supports this by stating that telehealth practice varies greatly from traditional practice and that practitioners should be equipped with relevant skills and competencies to succeed in its implementation.

The participants in this study have found that children with ASD and individuals with anxiety respond very well to telehealth. Clients who struggle with Anxiety tend to do well with having intervention sessions online as it proves less stressful to meet a stranger face-to-face as it allows the clients to feel as though they also have a sense of control, which many do not experience in in-person sessions.^39^ Although many people may feel that online therapy is not effective when it comes to children who have ASD, it has been proven to be just as effective as in-person therapy especially since it incorporates technology which many children with ASD are interested in.^40^ Therefore, the participants have used the evidence that is available to inform their practice concerning which clients are eligible for telehealth. Moreover, they have used their experiences, together with existing literature, to inform their practice.

Few participants mentioned how telehealth proved to be beneficial in improving assessment findings within the clients’ environments. Jacobs et al.^33^ mentioned that clients could benefit from tele-monitoring for accumulating assessments, with the use of asynchronous data, which participants mentioned they had incorporated methods such as utilising video conferencing, pictures and video recording to conceptualise assessment data for the therapist to utilise.

Multiple participants had expressed the limitations and challenges of building trust and personal relationships through online sessions; therefore, a hybrid telehealth method has been established, furthermore, Bhat^41^ has raised the same concerns which telehealth instils the limitations and difficulties experienced by the therapists end, thus stated in the study that greater enthusiasm and effort was required on virtual sessions for children specifically. Substantiating the rationality for utilising hybrid methods of alternating in-person and online sessions by the participants.

### Enhancing the potential of telehealth

Theme four addresses the need to enhance the potential of Telehealth. Participants of this study shared a common need for guidelines for telehealth in OT to be established, to increase their confidence in implementation. Rabe,^2^ after revision of the HPCSA Booklet 10 in December 2021, proposes that healthcare practitioners utilising telehealth services make use of this booklet which stipulates the guidelines to be followed, about record keeping, determining the eligibility of a client as well as providing a template of how practitioners can introduce Telehealth into their consultations. In a South African context, the lack of access to devices and digital literacy has been a barrier to the use of telehealth as mentioned by our participants throughout. Literature^42^ agrees that the lack of access to technology^43^ and digital literacy^43^ directly influences patient outcomes. Furthermore, Kobeissi et.al.^42^ encourage healthcare professionals to assist underprivileged populations by providing them with the technology devices needed to access these services.

Telehealth's integration into OT is gaining recognition for its potential to enhance patient outcomes and accessibility, particularly using technology and online platforms. Research emphasises that telehealth provides improved client safety and outcomes in therapy and encourages closer supervision from the healthcare professional.^44^ To prepare future OTs, educational initiatives are crucial, with studies advocating for intentional Telehealth integration in academic institutions^45^ as this allows telehealth training to be embedded within OT curricula. Finally, public engagement is essential, as studies show that telehealth clients are happy with their virtual healthcare and eager to use it again.^46^ These insights highlight the importance of a varied approach to promoting telehealth in OT.

## Limitations

A limitation of the current study consisted of the time allocated for the researchers to conduct the study and collect findings. Therefore, the number of participants was reduced, however, the researcher(s) were able to interview 10 participants despite the time limitation. Due to the time constraint, the researcher(s) were only able to interview private practice OTs utilising telehealth, the study may have benefited from OTs working in the public sector, however, they had occupied time schedules. A limitation of the study is that the research participants were all female OTs who reported the use of telehealth services. It is suggested that future studies perhaps focus on obtaining the views of male OT's using telehealth strategies. Another limitation was that the criteria consisted of the OTs requiring 3 months of experience utilising telehealth and that participants needed to be currently utilising telehealth, which both contributed to the limitation in the number of participants who could participate within the study.

## Implications to practice

Developing thorough training programmes for therapists which prioritise virtual assessment, engagement with clients, and technology for telehealth, while promoting ongoing professional growth, is important for enhancing the effective use of telehealth in OT. Furthermore, to overcome the difficulties of telehealth, continued research and development are essential for creating effective tools and evaluating long-term results. Future developments in telehealth services can be guided by frequent evaluation and monitoring, as well as input from therapists and clients. Increasing awareness and encouragement in the field of telehealth can be achieved through integrating it into student education and encouraging knowledge sharing among practitioners.

## Conclusion

The use of technology in primary health care continues to be a growing facet of healthcare, to provide healthcare services to larger populations. This led to an increase in health professionals utilising telehealth in practice, however, a lack of literature exploring the efficacy of telehealth in occupational therapy practice has been identified. This study, aimed to explore the experiences of occupational therapists who have made use of and currently utilise telehealth in their intervention. Contrary to the beliefs of other health professionals regarding occupational therapists making use of telehealth, the researchers found that the adaptability and creativity of occupational therapists have aided in the successful use of telehealth. This study explored and provided insight into the barriers experienced by both the participants and their clients influencing the use of telehealth including access to Wi-Fi or data and having devices and digital literacy to engage with telehealth successfully. Additionally, telehealth consultations led the participants to experience difficulty in completing assessments, as well as teaching the client the correct handling principles to apply during intervention sessions. However, the researchers also found that the use of telehealth allowed the participants to provide services to their clients without the geographic barriers and cost and time associated with travelling to the respective practices. Additionally, the techniques and adaptations applied by the participants provide the clients with the education needed to continue with treatment at home, as well as provide a sense of empowerment.

## Appendix 1

**Table 1.** Demographic information of the participants.

Demographics	Field of occupational therapy	Age	Gender	Years of experience in telehealth	Level of education
P1	Mental Health and Physical Health	27	Female	4 months	B. Occupational Therapy
P2	Burns, Plastic Surgery, Hand Therapy, and Neurology	62	Female	5	B. Occupational Therapy
P3	Mental Health OT & Eating Disorders.	63	Female	4	B. OT degree in Occupational Therapy
P4	Mental Health and Paediatrics	38	Female	5	BSC Occupational Therapy
P5	Acute psychiatric	29	Female	4	B. Occupational Therapy
P6	Adult Vocational Rehabilitation, Chronic Pain Management and Functional Capacity Evaluations.	37	Female	4	B. Occupational Therapy National Post Graduate Diploma in Vocational Rehabilitation
P7	Child and adult mental health including neurodevelopmental disorders	39	Female	5	BSc Occupational Therapy
P8	Psychosocial: Psychogeriatric Evaluations and Treatment and Vocational Rehabilitation	45	Female	5	B. Occupational Therapy
P9	Psychiatry with a focus on vocational rehabilitation, life skills development and cognitive rehabilitation.	30	Female	2	B. Occupational Therapy
P10	Mental Health		Female	6	B. Occupational Therapy M. Occupational Therapy

**Table 2.** Inclusion and exclusion criteria.

Inclusion criteria	Exclusion criteria
OTs must have at least 3 months of experience in using telehealth OTs must be actively employed or working in providing OT service OTs must still currently be using Telehealth in practice.	OTs who have not used telehealth as a service model in their practice or service.

## Appendix 2: Semi-structured Interview Guide

**SEMI-STRUCTURED QUESTIONNAIRE using open-ended questions**

Thank you so much for taking the time out of your day to take part in this study – we do appreciate it. Today we want to ask you a bit about your experiences with using telehealth and how you found this medium when doing intervention with your clients. We will keep your personal information confidential. Please do not feel pressured to provide a right or wrong answer. Do you have any questions before we begin?

**Question 1)** Could you describe the barriers (hospital-related factors, finances, training) that you experienced when using telehealth in your daily occupational practice

**Probe:** Please describe the circumstances that cause occupational therapists to not use telehealth in practice

**Probe:** Describe what limits your ability to use telehealth in the hospital setting

**Probe:** Describe whether you experienced any difficulties with clients in administering telehealth

**Question 2)** Please describe the aspects or factors that helped you in using telehealth as a strategy in occupational therapy practice

**Probe:** What have you found beneficial when administering telehealth

**Probe**: What resources that you have used helped you in using telehealth (for example, Wi-Fi or training)

**Probe:** Please describe whether your experience and exposure to telehealth has helped you to use telehealth in practice

**Question 3)** Could you describe your views on what telehealth is or how you see telehealth in the professions of occupational therapy

**Probe:** Please describe how you think telehealth contributes to evidence-based practice

**Probe:** Could you list some reasons you think telehealth would be more beneficial than in-person treatment in a South African context?

**Probe:** How has your telehealth-based treatment and client response differed from in-person treatment?

**Question 4)** If you were given the opportunity to improve the use of telehealth in occupational therapy practice, how would you do this?

**Probe:** How will you be able to ensure that more occupational therapists use telehealth in practice?

**Probe:** How can you make telehealth more effective in the occupational therapy practice (what would you require in terms of space, resources, training, making patients or clients confident in telemedicine as a strategy etc)

## References

[1] Sarsak H . Telerehabilitation services: A successful paradigm for Occupational Therapy Clinical Services? Int Phys Med Rehabil J 2020; 5. 10.15406/ipmrj.2020.05.00237

[2] Rabe M . Telehealth in South Africa: A guide for healthcare practitioners in primary care. S Afr Fam Pract 2022; 64. 10.4102/safp.v64i1.5533 PMC9257714 35792625

[3] Dehghani S Mirzakhany Araghi N Dehghani S , et al. The use of tele-occupational therapy for children and adolescents with different disabilities: systematic review of RCT articles. Med J Islam Repub Iran 2023. 10.47176/mjiri.37.17 PMC10134098 37123331

[4] Jansen van Vuuren J Okyere C Aldersey H . The role of occupational therapy in Africa: A scoping review. S Afr J Occup Ther 2020; 50. 10.17159/2310-3833/2020/vol50no3a2

[5] Hoel V von Zweck C Ledgerd R . Was a global pandemic needed to adopt the use of telehealth in occupational therapy? Work 2021; 68: 13–20. 33337401 10.3233/WOR-205268

[6] Dahl-Popolizio S Carpenter H Coronado M , et al. Telehealth for the provision of occupational therapy: reflections on experiences during the COVID-19 pandemic. Int J Telerehabil 2020; 12: 77–92. 33520097 10.5195/ijt.2020.6328 PMC7757642

[7] Breeden LE Tyger H Reckers AM , et al. An examination of occupational therapy telehealth service delivery among novice users during the COVID -19 pandemic. Int J Telerehabil 2023; 15. 10.5195/ijt.2023.6544 PMC10687948 38046553

[8] Feldhacker DR Jewell VD Le Sage SG , et al. Telehealth interventions within the scope of occupational therapy practice: a systematic review. The Amer J of Occup T 2022; 76: 7606205090. 10.5014/ajot.2022.049417 36332197

[9] Kayyali R Hesso I Mahdi A , et al. Telehealth: misconceptions and experiences of healthcare professionals in England. Int J Pharm Pract 2017; 25. 10.1111/ijpp.12340 28261891

[10] Fisher K Magin P . The telehealth divide: health inequity during the COVID-19 pandemic. Fam Pract 2022; 39. 10.1093/fampra/cmab173 PMC9383174 34964886

[11] Gurupur VP Miao Z . A brief analysis of challenges implementing telehealth in a rural setting. Mhealth 2022; 8. 10.21037/mhealth-21-38 PMC9014233 35449506

[12] Malatji MT . Rural development outcomes and policies in South Africa’s Limpopo Province. Johannesburg, South Africa: UNISA, 2020. http://hdl.handle.net/10500/26719 .

[13] Langarizadeh M Moghbeli F Aliabadi A . Application of ethics for providing telemedicine services and information technology. J Acad Med Sci 2017; 71. https://doi.org/10.5455%2Fmedarh.2017.71.351-355 10.5455/medarh.2017.71.351-355 PMC5723167 29284905

[14] Townsend BA Scott RE . The development of ethical guidelines for telemedicine in South Africa. S Afr J Bioeth Law 2019; 12. https://hdl.handle.net/10520/EJC-171924bfb2

[15] Haleem A Javaid M Singh RP , et al. Telemedicine for healthcare: capabilities, features, barriers, and applications. Sens Int 2021; 2: 100117. 34806053 10.1016/j.sintl.2021.100117 PMC8590973

[16] Cribb A Entwistle V Mitchell P . What does ‘quality’ add? Towards an ethics of healthcare improvement. J Med Ethics 2020; 46: 118–122. 31732680 10.1136/medethics-2019-105635 PMC7035683

[17] Ashburner J Vickerstaff S Beetge J , et al. Remote versus face-to-face delivery of early intervention programs for children with autism spectrum disorders: perceptions of rural families and service providers. Res Autism Spectr Disord 2016; 23: 1–14.

[18] Zylstra SE . Evidence for the use of telehealth in pediatric occupational therapy. J Occup Ther Sch Early Interv 2013; 6: 326–355.

[19] Little LM Pope E Wallisch A , et al. Occupation-based coaching by means of telehealth for families of young children with autism spectrum disorder. Am J Occup Ther 2018; 72: 1–7. 10.5014/ajot.2018.024786 29426380

[20] Angell AM Carreon ED Akrofil JNS , et al. Challenges and facilitators to telehealth occupational therapy for autistic children during COVID-19. OTJR (Thorofare N J) 2023; 43: 513–522. 36597578 10.1177/15394492221142597 PMC9816621

[21] Little LM Wallisch A Pope E , et al. Acceptability and cost comparison of a telehealth intervention for families of children with autism. Infants Young Child 2018; 31: 275–286.

[22] Sánchez-Guarnido AJ Domínguez-Macías E Garrido-Cervera JA , et al. Occupational therapy in mental health via telehealth during the COVID-19 pandemic. Int J Environ Res Public Health 2021; 18: 7138. 34281072 10.3390/ijerph18137138 PMC8297153

[23] Grant C Jones A Land H . What are the perspectives of speech pathologists, occupational therapists and physiotherapists on using telehealth videoconferencing for service delivery to children with developmental delays? A systematic review of the literature. Aust J Rural Health 2022; 30: 321–336. 35157335 10.1111/ajr.12843 PMC9303186

[24] Hunter D McCallum J Howes D . Defining exploratory-descriptive qualitative (EDQ) research and considering its application to healthcare. 2019.

[25] Naderifar M Goli H Ghaljaie F . Snowball sampling: a purposeful method of sampling in qualitative research. Strides Dev Med Educ [Internet] 2017; 14(. https://sdme.kmu.ac.ir/article_90598.html

[26] Malterud K Siersma VD Guassora AD . Sample size in qualitative interview studies: guided by information power. Qual Health Res 2016; 26: 1753–1760. 26613970 10.1177/1049732315617444

[27] Braun V Clarke V . Using thematic analysis in psychology. Qual Res Psychol 2006; 3: 77–101. http://eprints.uwe.ac.uk/11735

[28] Krefting L . Rigour in qualitative research: the assessment of trustworthiness. Am J Occup Ther 1991; 45: 214–222. 2031523 10.5014/ajot.45.3.214

[29] Edirippulige S Armfield N . Education and training to support the use of clinical telehealth: a review of the literature. J Telemed Telecare 2017; 23: 273–282. 26892005 10.1177/1357633X16632968

[30] Proffitt R Cason J Little L , et al. Stimulating research to advance evidence-based applications of telehealth in occupational therapy. OTJR: Occup Particip Health 2021; 41: 153–162. 10.1177/15394492211011433 PMC9491013 33926321

[31] Khoza-Shangase K Moroe N . South African hearing conservation programmes in the context of Tele-Audiology: A scoping review. South Afr J Commun Disord 2020; 67. doi:10.4102/sajcd.v67i2.670 PMC7136824 32129657

[32] Aruleba K Jere N . Exploring digital transforming challenges in rural areas of South Africa through a systematic review of empirical studies. Sci Afr 2022; 16. doi:10.1016/j.sciaf.2022.e01190

[33] Jacobs K Cason J McCullough A . The process for the formulation of the international telehealth position statement for occupational therapy. Int J Telerehabil 2015: 21–32. doi:10.5195/ijt.2015.6163 PMC4985276 27563380

[34] Houser SH Flite CA Foster SL . Solutions for challenges in telehealth privacy and security [Internet]. J AHIMA 2022 [cited 2024 Aug 24]. https://journal.ahima.org/page/solutions-for-challenges-in-telehealth-privacy-and-security

[35] McCoyd JL Curran L Candelario E , et al. “There is just a different energy”: changes in the therapeutic relationship with the telehealth transition. Clin Soc Work J 2022; 50: 325–336. 35493775 10.1007/s10615-022-00844-0 PMC9035977

[36] Sundstrup E Meng A Ajslev JZ , et al. New technology and loss of paid employment among older workers: prospective cohort study. Int J Environ Res Public Health 2022; 19: 7168. 35742416 10.3390/ijerph19127168 PMC9222591

[37] Wamala DS Augustine K . A meta-analysis of telemedicine success in Africa. J Pathol Inform 2013; 4: 6. 23858382 10.4103/2153-3539.112686 PMC3709418

[38] Mahtta D Daher M Lee MT , et al. Promise and perils of telehealth in the current era. Curr Cardiol Rep 2021; 23. doi:10.1007/s11886-021-01544-w PMC8283748 34269884

[39] Rose NN Ishak AS Abdullah S , et al. Online solution focused therapy counselling for clients with mental health issues (anxiety and depression). In: AIP conference proceedings, 2023. doi:10.1063/5.0127824

[40] Nayer N Vijay V Sudheer A , et al. Are Online Interventions Effective for My Child with ASD? A Review of Online Interventions for Pre-Primary and Primary Aged Children with ASD during the COVID-19 Pandemic. Int J Sci Res 2023; 11: 293–298. https://www.researchgate.net/publication/374381121_Are_Online_Interventions_Effective_for_My_Child_with_ASD_A_Review_of_Online_Interventions_for_Pre-Primary_and_Primary_Aged_Children_with_ASD_during_the_COVID-19_Pandemic

[41] Bhat A Su W-C Cleffi C , et al. A hybrid clinical trial delivery model in the COVID-19 ERA. Phys Ther 2021; 101. doi:10.1093/ptj/pzab116 PMC8135561 33909902

[42] Kobeissi MM Hickey JV . An infrastructure to provide safer, higher-quality, and more equitable telehealth. Jt Comm J Qual Patient Saf 2023; 49: 213–222. 36775714 10.1016/j.jcjq.2023.01.006 PMC9839454

[43] US Department of Health and Human Services, Health Resources and Services Administration . Health equity in telehealth [Internet]. 2022 Jun 3 [cited 2023 Jan 18]. https://telehealth.hhs.gov/providers/health-equity-in-telehealth/

[44] Marwaa MN Kristensen HK Guidetti S , et al. Physiotherapists’ and occupational therapists’ Perspectives on Information and Communication Technology in stroke rehabilitation. PLOS ONE 2020; 15. doi:10.1371/journal.pone.0236831 PMC7454973 32857781

[45] Patterson A Feldhacker DR Greiner BS , et al. Telehealth and Occupational Therapy Education. J Occup Ther Educ 2021; 5. doi:10.26681/jote.2021.050406

[46] Kennedy A . Millennials and zoomers vs. boomers: How different generations seek out and choose healthcare providers [Internet]. PressGaney 2022 [cited 2024 Aug 25]. https://info.pressganey.com/press-ganey-blog-healthcare-experience-insights/millennials-and-zoomers-vs-boomers-how-different-generations-seek-out-and-choose-healthcare-providers

